# Improving the quality of image generation in art with top-*k* training and cyclic generative methods

**DOI:** 10.1038/s41598-023-44289-y

**Published:** 2023-10-18

**Authors:** Laura Vela, Félix Fuentes-Hurtado, Adrián Colomer

**Affiliations:** 1 Universidad Internacional de Valencia (VIU), Calle Pintor Sorolla, 21, 46002 Valencia, Spain; 2 KNODIS Research Group, Universidad Politécnica de Madrid, Madrid, Spain; 3 Departamento de Sistemas Informaticos, Universidad Politécnica de Madrid, Madrid, Spain; 4 Instituto Universitario de Investigación en Tecnología Centrada en el Ser Humano, HUMAN-tech, Universitat Politècnica de València, Valencia, Spain; 5 ValgrAI-Valencian Graduate School and Research Network for Artificial Intelligence, Valencia, Spain

**Keywords:** Computer science, Computational science

## Abstract

The creation of artistic images through the use of Artificial Intelligence is an area that has been gaining interest in recent years. In particular, the ability of Neural Networks to separate and subsequently recombine the style of different images, generating a new artistic image with the desired style, has been a source of study and attraction for the academic and industrial community. This work addresses the challenge of generating artistic images that are framed in the style of pictorial Impressionism and, specifically, that imitate the style of one of its greatest exponents, the painter Claude Monet. After having analysed several theoretical approaches, the Cycle Generative Adversarial Networks are chosen as base model. From this point, a new training methodology which has not been applied to cyclical systems so far, the top-*k* approach, is implemented. The proposed system is characterised by using in each iteration of the training those *k* images that, in the previous iteration, have been able to better imitate the artist’s style. To evaluate the performance of the proposed methods, the results obtained with both methodologies, basic and top-*k*, have been analysed from both a quantitative and qualitative perspective. Both evaluation methods demonstrate that the proposed top-*k* approach recreates the author’s style in a more successful manner and, at the same time, also demonstrate the ability of Artificial Intelligence to generate something as creative as impressionist paintings.

## Introduction

Art as a representation of the human being and, specifically, the visual arts as a manifestation of ideas, feelings, way of life and social context, have been present throughout the humankind history. From the ancestral cave paintings found in most continents, to the different artistic styles that have prevailed during each historical period, painting has been for centuries the main means of documenting reality and expressing emotions.

Generating good artistic samples using deep learning techniques is of significant importance for several reasons. Firstly, it enables the exploration of new possibilities in artistic creation by leveraging the capabilities of artificial intelligence. This intersection between AI and art opens up avenues for novel and innovative artistic expressions, pushing the boundaries of traditional artistic practices. Moreover, generating high-quality artistic samples holds the potential to democratize art. By automating the creation process, AI-based systems can make art more accessible to a wider audience, irrespective of their artistic skills or training. This inclusivity fosters creativity and allows individuals to engage with art in meaningful ways, promoting cultural appreciation and expression. Furthermore, Deep Learning models can capture the essence of renowned artists, such as Claude Monet, and recreate their styles with remarkable accuracy. This preservation of artistic heritage not only pays homage to past masters but also allows contemporary artists to build upon their legacy, leading to a continuous evolution of artistic practices^1^.

As a result, the distance, a priori abysmal, between art and Artificial Intelligence, has been narrowing in recent years as human beings have gone deeper into specific branches of AI such as Expressive Artificial Intelligence (EAI). This branch of AI focuses on the development of intelligent algorithms capable of generating works of art. The essence of EAI is substantially different from other research fields, since artistic ability or creativity represents a subjective quality; that is, it does not allow to define a theoretical set of rules that evaluate the algorithm’s output. Therefore, the design of techniques capable of establishing an interaction between AI and art has been a challenge for the scientific community in recent years, envisioning the possibility of developing the artistic world through the use of this branch of computer science.

In relation to AI applied to the visual arts, numerous works have been carried out in recent years, among which the “Aaron project” initiated by Harold Cohen in 1973 and which is currently still in progress, stands out. Aaron^2^ is an algorithm that uses an AI engine combined with a database of artistic techniques capable of generating its own creative version from a given canvas.

Beyond the generation of new artistic images based on the concept of creativity, much of the efforts of the scientific community in recent years have focused on the generation of images that mimic a given artistic style. In particular, the evolution of Neural Networks during the last decades has meant a great advance in this aspect. Thus, two essential elements have been considered in any work: style and content. Style is conceived as the way in which the artist expresses his ideas on the canvas or any other surface. On the other hand, we refer to the concept of the content of a work of art as its semantic part, that is, the representation of the objects that can be recognised in its style^3^.

In 2018, a work of art made by an AI algorithm was sold at auction for the first time. The piece in question was a portrait of Edmond Belamy, inspired by 18th century aesthetics. The project was developed by a French multidisciplinary collective called Obvious formed by Pierre Fautrel, Hugo Caselles-Dupré and Gauthier Vernier; artist, computer scientist and economist, respectively. The methodology used was a neural network whose training was performed using a dataset of 15,000 portraits painted between the fourteenth and twentieth centuries and from which the characteristic features of the style of the particular period were extracted. The context outlined so far must be completed with a mention of the French painter Claude Monet, an essential figure in this work since his works and his unique style will be essential in the approach we will take to the EAI. Claude Monet (1840–1926) is considered the greatest exponent of the impressionist movement. His work is characterised by a palpable obsession to express emotions through the effect that light produces on nature. After going into exile in Algeria to avoid military service, his stays in Europe led to his growing interest in the study of light at different times or in different seasons. Gradually, he accentuated the luminous effects to the point of sometimes blurring forms or even fusing them together^4^.

**Figure 1:**
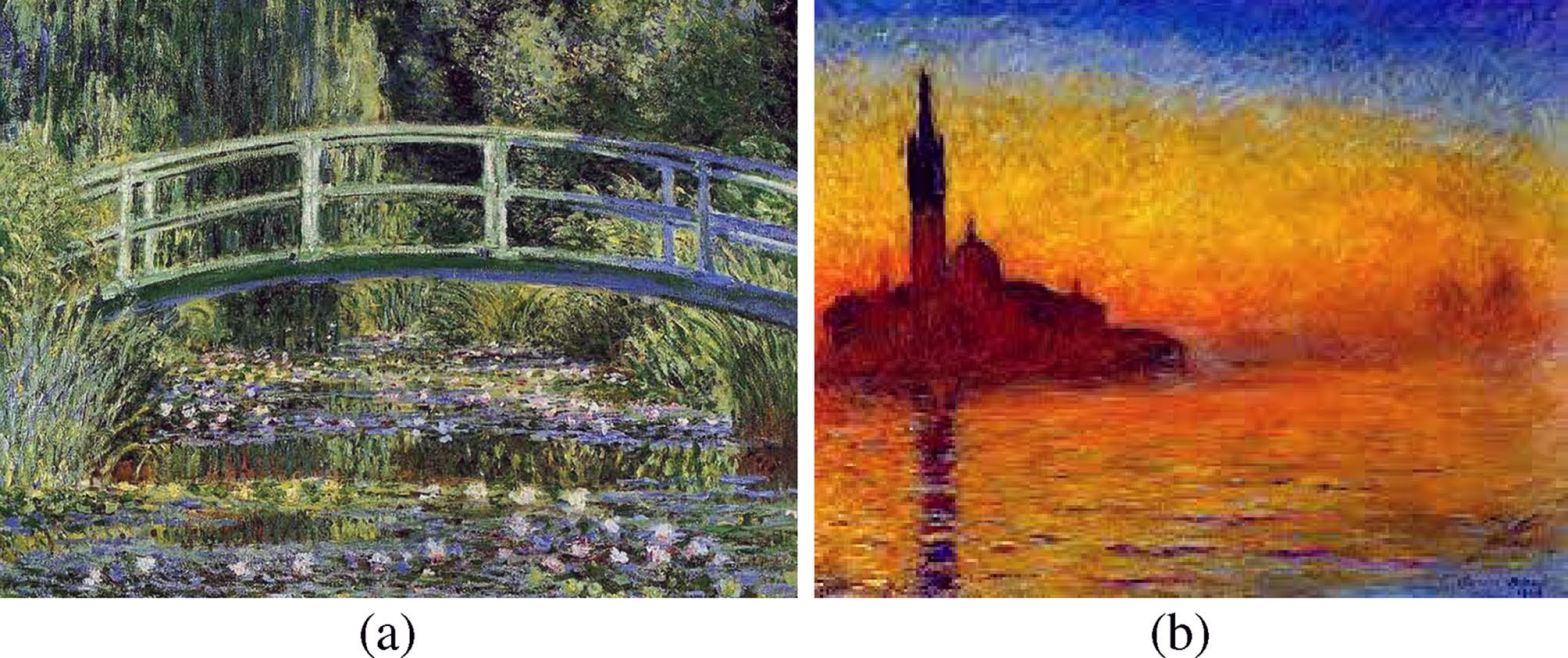
(**a**) “Water Lily Pool an Japanese Bridge” (1899); (**b**) “Twilight in Venice” (1912).

While the most common among his contemporaries was to paint in seclusion in studios or galleries, Claude Monet’s art was based on the opposite. His intention was not to capture on canvas the idea of a perfect landscape, but to faithfully reflect the nature that surrounded him. Therefore, all his works were made outdoors, becoming one of the forerunners of painting *en plein air* or outdoors. Furthermore, we can conclude that his oil painting goes beyond the conditions of the moment; it does not follow the prevailing line of romanticism, classicism or realism, but places its focus on the senses and on nature^5^. Among his most important works we can highlight the ones shown in Fig. 1.

This very characteristic style of Monet’s works has been the subject of study by the scientific community repeatedly, trying to generate images that mimic the artist’s style. The article *“Creating art with deep learning”*^6^ published in December 2018 applies and compares different types of DL architectures to transfer the style from an artistic painting to any photograph. On the other hand, May 2019 sees the publication of the paper *“Art2Real: Unfolding the Reality of Artworks via Semantically-Aware Image-to-Image Translation”*^7^, in which various DL architectures are used to generate landscape snapshots from works by different artists. Recently, the study carried out by Kotovenko *et al*. and published in March 2020^8^ stands out because it develops a Neural Network architecture that aims to transform any input image into works painted by different well-known artists (Kandinsky, Van Gogh, Gauguin, Cézanne, Picasso and Monet, among others). In another recent work^9^, Vivek Ramanujan *et al*. (2021) propose a new approach to style transfer that uses parameterised brushstrokes to represent the style of an image.

In addition to these works, some other recent works have explored the use of stable diffusion models for image style transfer^10,11^.

The importance of our study lies in two key aspects:

- *Novel training methodology* applied to art generation: We propose a novel training methodology for Cycle GAN models based on the top-*k* approach, inspired by the work of^12^. This unique training scheme has the potential to enhance the convergence and performance of the model, leading to improved results in terms of loss functions and image quality.
- *Quantitative and qualitative evaluation by an expert group*: We provide a comprehensive evaluation of our proposed methodology from both quantitative and qualitative perspectives, the latter performed by an Expert Group.

The remainder of the paper follows the following structure: next Section provides an overview of the Related work, then, we detail our Results, following, we provide a comprehensive Discussion, and finally, we present the methods employed in the study.

## Related work

### Rendering-based precedents

Prior to the analysis of AI techniques used for the generation of artistic images, it is necessary to mention an area of knowledge that is included within the graphic arts, known as Non Photorealistic Rendering (NPR)^13^. This area encompasses all those techniques that, starting from a real image, try to simulate a non-photorealistic representation, for example, imitating the style of a pencil drawing, a watercolor or a color canvas^14^.

The main limitation of the NPR methodology as a whole is the lack of flexibility of the algorithms themselves. Thus, these algorithms specialise in transferring a specific style, not being generalizable to other styles. Therefore AI, and in particular Convolutional Neural Networks (CNNs), offer different strategies to overcome this limitation.

### Neural style transfer

In order to overcome the restrictions exposed in the previous section, it is particularly relevant the experimental study about Convolutional Neural Networks conducted in 2015 and led by Gatys et al. in which it became clear that CNNs were able to extract information and style features from an image based on pattern detection. The key in this work was the discovery that an image produces both content and style activations, as well as the ability to differentiate between them.

Thus, the application of style to a given image is achieved by iteratively updating the weights relative to both content and style present in the cost function. It was a disruptive work since it presented no restrictions between different styles and no labelled training set was needed. This study initiated the current known as Neural Style Transfer (NST), whose technique establishes a starting point with two images: “Content Image”, which includes the semantic part, i.e. what is represented in it (a beach, a tree, several people); and the “Style Image”, which is the one containing the textures we aim to achieve. The objective is to generate a new image, “Generated Image”, which has the semantic information of the “Content Image” but with the style of the “Style Image”^15^. In this line, the study carried out later by Johnson et al. included the open source implementation of the system proposed by Gatys et al., which consisted of training a VGG-19 as proposed in the initial study. The results obtained, similar to those of Gatys et al., were limited by the lack of a method to ensure that, in addition to achieving style transfer in the Generated Image, the semantic content of the initial image was preserved. Moreover, the computational cost required was high^16^.

In this sense, it is worth mentioning the work carried out later by the team of Dimitry Ulyanov et al. who proposed the use of a Feed-Forward Convolutional Network for image pattern recognition and subsequent classification. The main differences of this work in comparison with the one proposed by Gatys et al. are mainly based on the use of more efficient loss functions which involves a shorter processing time. Thus, they managed to considerably reduce the time cost of the whole process^17^. On the other hand, Rujie Yin et al. published in January 2016 a work in which they attempted to perform texture transfer with the aim of preserving, as much as possible, the semantic information of the starting image. Although they also used a VGG-19 as a starting point, the key to their system was the segmentation of the “Content Image” into several parts, each corresponding to the same object or semantic field. Once this fragmentation was performed, texture transfer was carried out individually on each of these parts, finally proposing the union of all of them and thus ensuring a much more accurate and higher resolution transfer^18^.

Even though the quality of style transfer using NST has been gradually increasing as a result of the studies carried out in this regard, the present work has not opted for this technique for different reasons. On the one hand, the computational cost of training the network and its subsequent implementation is too high. On the other hand, as we have previously analysed, such an approach starts from a single style image (“Style Image”), which greatly reduces the possibilities in terms of transferring the impressionist features that define Claude Monet’s work.

Likewise, the starting point of the present work is not a single picture of the painter, but a complete dataset with a large part of his works in which a wide range of features and textures that characterise his style are shown and that, subsequently, we will apply on the input image. This leads us to reject the use of techniques based on the use of a single image.

Some of the most recent works include that of Liu et al.^19^, who learn spatial attention score from both shallow and deep features by an adaptive attention normalization module (AdaAttN). An et al.^20^ alleviate content leak by reversible neural flows and an unbiased feature transfer module (ArtFlow).

Recent research efforts have explored contrastive learning approaches for image style transfer, such as language-driven artistic style transfer (LDAST)^21^ utilizing contrastive language visual artist (CLVA) for consistent content structures and analogous style patterns, as well as zero-shot contrastive loss for diffusion models^22^ enabling high-quality image generation without additional fine-tuning or auxiliary networks, surpassing existing methods in tasks like image style transfer, image-to-image translation, and manipulation.

### Generative adversarial networks

Generative Adversarial Networks (GANs) have been gaining popularity in recent years among the scientific community, since they provide additional value over traditional models consisting of the ability to create content. The *vanilla* version of the GANs, as described in the study by Goodfellow et al. is based on the definition of a model in which there are two antagonistic networks: a Generator and a Discriminator^23^.

This *vanilla* version of GANs presents several difficulties in terms of network training: (i) “collapse mode”, consisting in the fact that the Generator, once it has verified that a set of images is useful for “fooling” the Discriminator, tends to always create the same set, losing variability in the output images and, consequently, losing “creativity”^24^; (ii) “oscillating loss”, meaning that during the training of GANs, the loss of the Generator and the Discriminator can become excessively large, without converging; and (iii) “lack of ex-post control” over the content of the generated images. This may result in the generated content not meeting the user’s requirements, or in the quality of the generated images being unsatisfactory. In order to overcome the aforementioned obstacles of the *vanilla* version, several versions of GANs have been designed during the last decade.

First, the so-called *Wasserstein GAN* (WGAN)^25^ and its successor, the *Wasserstein GAN - Gradient Penalty* (WGAN-GP)^26^, are improvements to the *vanilla *version that seek to increase stability by training the model and reducing the “collapse mode” problem. To this end, both replace the binary cross-entropy loss function with a loss function that correlates with the quality of the generated images.

On the other hand, *Conditional GANs* (CGANs)^27^ address the problem of poor control over the content of the *vanilla *version. For this purpose, they use external information to guide the image generation process. Thus, a CGAN model always combines a basic GAN and a source of external information, such as discrete class labels, text descriptions, semantic maps, conditional images, object masks or attention maps, to better control the content of the generated image.

**Figure 2:**
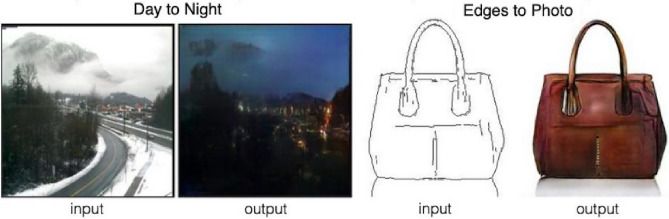
Example of the pix2pix model. Image-to-Image Translation with CGANs^25^.

Using CGANs as a basis, the study known as pix2pix^28^ has been of great interest to the scientific community since its publication in 2016. The key to this model is the transformation of a source image, belonging to one domain *X*, to an output image belonging to another domain *Y*. This study shows that the model is able to translate, for example, from the domain of images taken during the day to images taken at night (“*Day to Night*”, Fig. 2), or from images where only edges are shown to a domain where complete images are shown (“*Edges to Photo*”, Fig. 2).

In view of the results obtained with the pix2pix model, it could seem a priori a good solution to the challenge posed in the present work, as it allows the conversion of photographs (domain *X*) to Monet frames (domain *Y*). However, the pix2pix model has the conceptual obstacle that it is based on a supervised learning approach, i.e. training the model requires labelled pairs of examples. In other words, we would need to have the same element in both domains in order to subsequently label them and introduce them into the training process. This requirement is impossible in our case, given that we do not have photographs corresponding to each of the landscapes that Monet has recreated in his works. Likewise, we are not able to obtain a work that would be painted by the author from snapshots taken today.

This limitation is the starting point for the study of^29^, published a few months after pix2pix. This work constitutes a turning point in the GANs paradigm since it introduces the concept of *Cycle-Consistent Adversarial Networks* (Cycle GANs).

This approach allows cross-domain conversion without requiring direct correspondence between individual images from each domain. All that is needed are labelled images belonging to each of the domains, but these images do not need to be paired. In the example illustrated previously in 2, it would not be necessary to have pairs of photographs of the same landscape by day and by night, but only photographs of any landscape taken by day (for domain *X*) and photographs of any other landscape taken at night (for domain *Y*).

Beyond the above advantage, Cycle GAN outperforms the pix2pix approach given that it offers bidirectional conversions^24^, i.e. it allows an image to be converted from the *X* domain to the *Y* domain and vice versa. In our particular case, the use of a Cycle GAN model makes it possible to convert photographs to Monet paintings and, in turn, Monet paintings to photographs. In this way, we can both recreate the landscape that Monet actually saw when he produced his works, and generate works of art from today’s reality just as this impressionist painter would have done.

For the reasons given so far, the Cycle GAN model is the one chosen as the basis for the development of the present work. The architecture of this model is composed by four Neural Networks: two Generators (*G* y *F*) and two Discriminators ($D_X$ y $D_Y$). *G* transforms the images from the domain *X* to the domain *Y*. On the other hand, *F* transforms the images from the domain *Y* to the domain *X*. $D_Y$ tries to discern whether the input image is a real image or a fictitious image that tries to resemble the domain *Y*. On the other hand, $D_X$ tries to discriminate whether an input image is a real image or a *fake* image that tries to resemble the domain *X*^29^.

**Figure 3:**
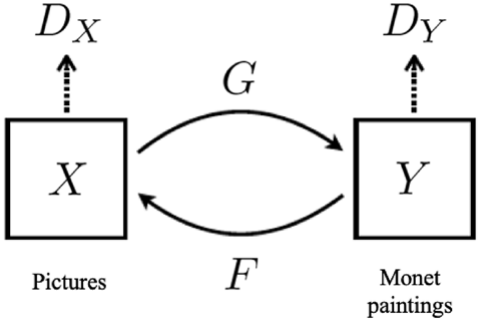
Cycle GANs scheme applied to the present work—image adapted from^26^.

Applying this scheme to the challenge posed in the present work, the image domains *X* and *Y* correspond to photographs and Monet paintings, respectively. Therefore, as we can see in Fig. 3, the Generator *G* tries to convert photographs into Monet paintings, and the Generator *F* to transform works by the artist into photographs. On the other hand, the Discriminator $D_Y$ tries to discern when the *input* image is really a work painted by Monet or a fictitious image generated by *G*. Finally, the Discriminator $D_X$ decides whether the *input* is really a photograph or an image generated by *F* from a painting.

Some other works leveraging GAN architectures include those of Chen et al.^30^, who apply internal-external scheme to learn feature statistics (mean and standard deviation) as style priors (IEST); or Zhang et al.^31^, that learn style representation directly from image features via contrastive learning to achieve domain enhanced arbitrary style transfer (CAST). Batziu et al.^32^ introduces a novel approach that combines CycleGANs with Fast and Adaptive Bidimensional Empirical Mode Decomposition (FABEMD) to effectively adopt a specific artist’s style on images. The proposed method modifies the cycle-consistency loss to incorporate texture information by estimating corresponding Bidimensional Intrinsic Mode Functions (BIMFs), and experimental results show that this approach outperforms state-of-the-art methods in artistic Neural Style Transfer applications.

### Top-*k* GANs approach

Continuing with the advances developed by the scientific community in relation to GANs, it is essential to mention the work carried out by Sinha et al. and published in October 2020, in which an innovative methodology for training GANs was addressed.

**Figure 4:**
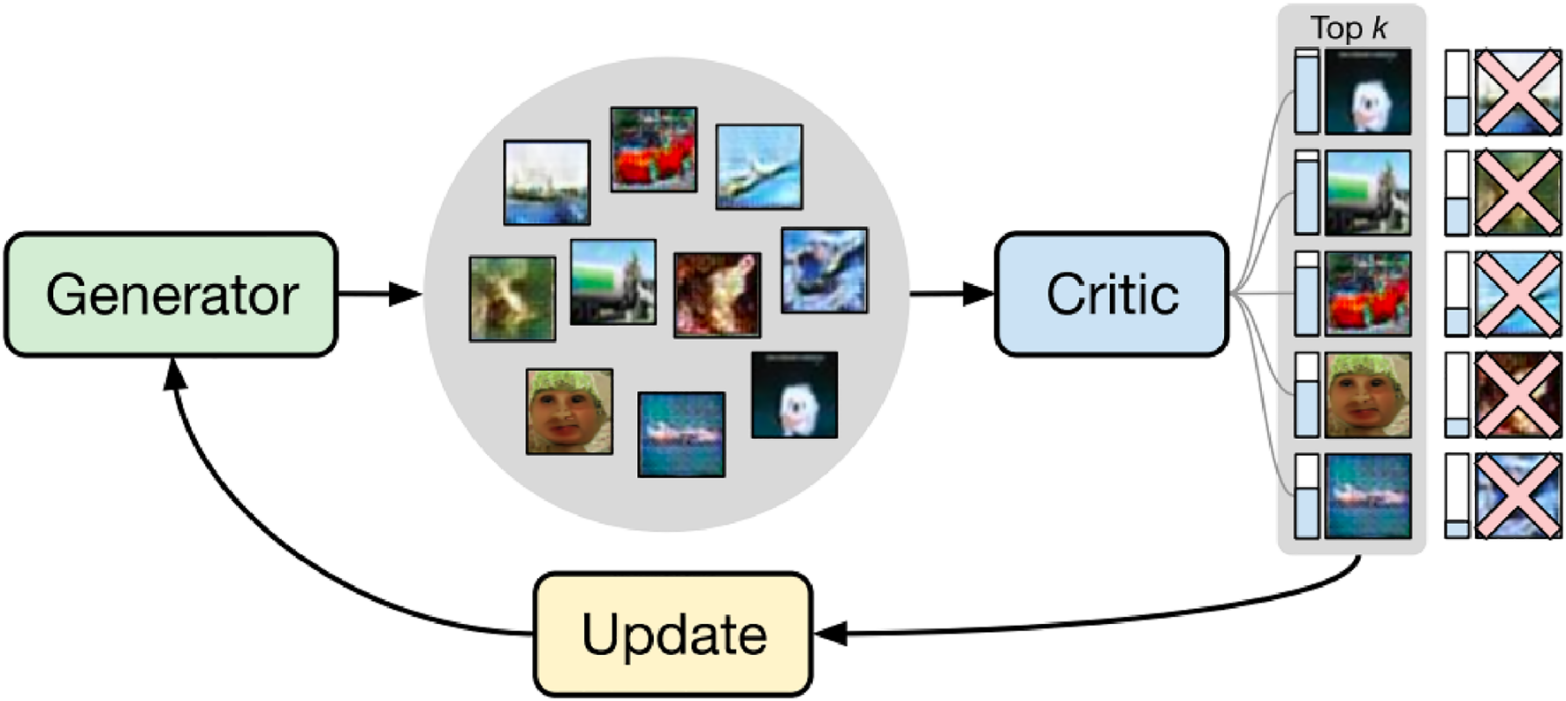
Training process in the top-*k* GANs approach^12^.

This study argued that in order to update the Generator weights in a GAN, not all the dummy images created by the Generator should be used, but only the images that have been able to best “fool” the Discriminator. In other words, only the *k* most realistic *fake* images, which have produced the lowest *Adversarial Loss* would be used to update the weights of the Generator. The authors demonstrate in their work that this modification substantially improves the results without increasing the computational cost^12^. In this way, as shown in Fig. 4, from the *batch* of images produced by the Generator, only those that, as indicated by the blue bar to the left of each illustration, are most similar to the real images, are used for the weight update process.

From the experiment performed with different variants of GANs (*vanilla GAN*, CGAN, WGAN and WGAN-GP), it is shown that the effectiveness of the top-*k* approach lies in discarding those images created by the Generator that are not really similar to the output set. In this way, the analyses carried out show that including such images could cause the gradient to move in the “wrong” direction, thus hindering convergence in training. To quote from the *abstract* of the above-mentioned report^12^:

[...] when gradient updates are computed using the worst-scoring batch elements, samples can actually be pushed further away from their nearest mode.

To date, no previous works have taken advantage of the promising top-*k* approach applied to train a cyclic structure like the aforementioned Cycle GANs. The present work develops this methodology and evaluates its results from a quantitative and qualitative perspective.

### Transformer and diffusion models

Besides CNN and GAN architectures, visual transformer and diffusion models based in transformers have also been used recently for style transfer tasks. Wu et al.^33^ perform content-guided global style composition by a transformer-driven style composition module (StyleFormer). Deng et al.^34^ propose a transformer-based method (StyTr2) to avoid the biased content representation in style transfer by taking long-range dependencies of input images into account. Even more recently, diffusion models have proven themselves as a very good alternative to deal with style and content separately when producing images^35^. In the art scope^10^, propose an Iterative Latent Variable Refinement method applied to Denoising Diffusion Probabilistic Models (DDPM) process to generate high-quality images based on a reference image. Very recently, Zhang et al.^11^ proposed a method able to learn the artistic creativity directly from a single painting to then guide the synthesis. Another work leveraging Difussion models is that of Chang et al.^36^, that introduce InST, an inversion-based style transfer method that learns the artistic style directly from a single painting, enabling efficient and accurate transfer without complex textual descriptions, achieving high-quality results across various artists and styles.

## Artificial intelligence and art

To sum up, there are two recent reviews dealing with artistic image generation^37^ and artistic image style transfer^1^. The former provides a comprehensive analysis of the utilization of Artificial Neural Networks and Deep Learning in the Visual Arts. It covers various applications such as prediction, classification, evaluation, generation, and identification in fields including photography, pictorial art, 3D modeling, video games, architecture, and comics. The latter explores the field of AI-generated art, discussing various deep neural network architectures and models utilized in its creation. From classic convolutional networks to cutting-edge diffusion models, it provides an overview of their structures and workings; and showcases milestones in AI-generated art, from DeepDream to recent developments like Stable Diffusion and DALL-E 2, highlighting their strengths and limitations. Both works demonstrate the ongoing evolution and substantial growth of Artificial Neural Networks in the Visual Arts, the remarkable progress made by deep neural networks in a short period of time and showcase the intersection of art and computer science in this field.

## The proposed approach

In this section, we will introduce the data employed in the study. We will also review the proposed system based on a Cycle GAN architecture, i.e. it is composed by two Generators (*G* y *F*) and two Discriminators ($D_X$ y $D_Y$) but, unlike what the scientific community has been developing, it is trained following the top-*k* approach proposed by Sinha et al.^12^.

### Data

In order to develop and to evaluate the proposed learning methodology, the cycle_gan/monet2photo dataset^38^ provided by TensorFlow has been used.

**Table 1:** Summary table of the cycle_gan/monet2photo dataset.

Set	Subset	Content	Size (pixels)
A	Train A	6.287 pictures	256 $\times$ 256
	Test A	751 pictures	256$\times$ 256
B	Train B	1.072 Monet’s	256 $\times$ 256
	Test B	121 Monet’s	256 $\times$ 256

As shown in Table 1, it is composed by 7038 photographs and 1193 pieces of art by Monet.

### Proposed architecture

**Figure 5:**
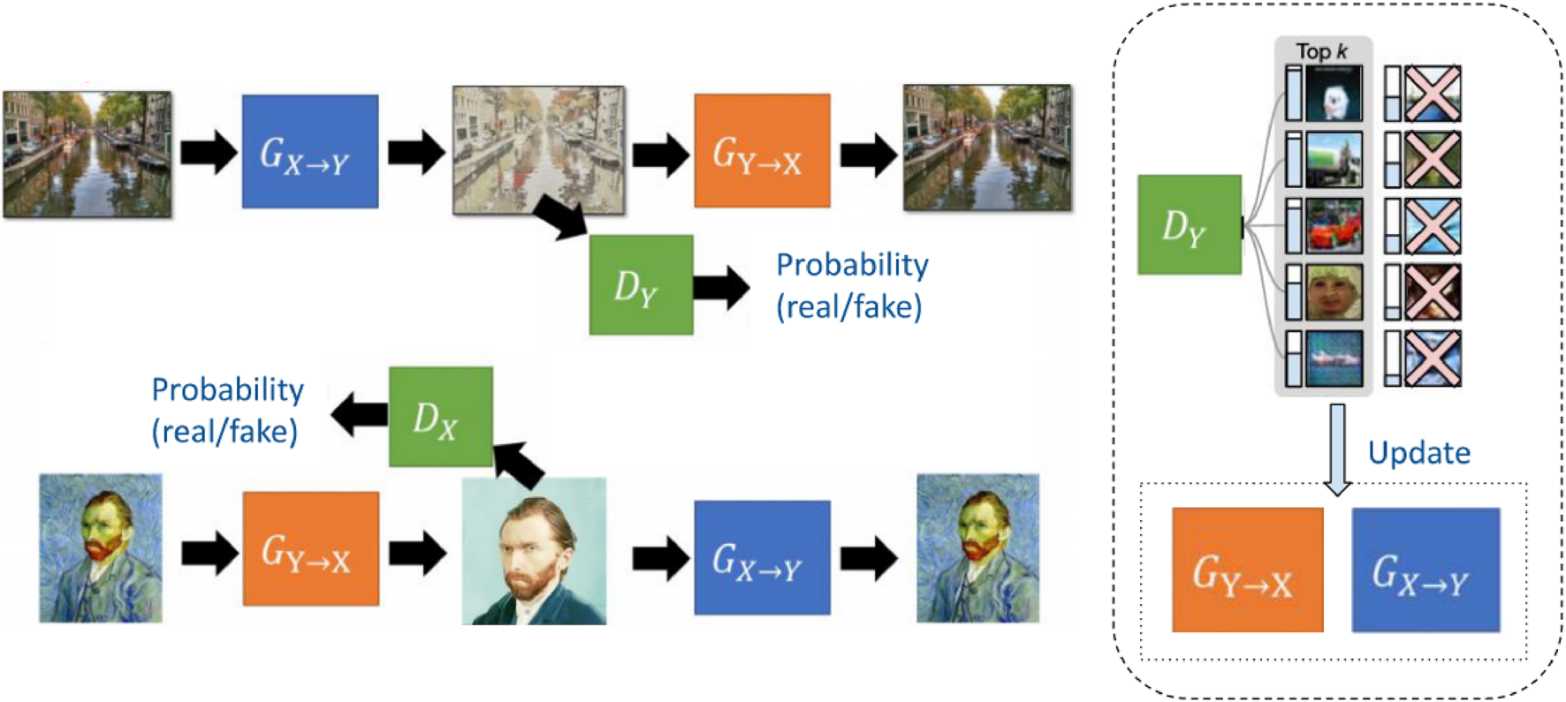
Proposed approach. We implement the CycleGAN model with a PatchGAN discriminator and train it using the *top-k* approach.

In this work, we leverage the CycleGAN architecture^38^ and train it using the *top-k* method^12^, as shows Fig. 5. Concretely, our generative network architecture is adapted from Johnson et al.^16^, known for its remarkable performance in neural style transfer and super-resolution tasks. This architecture consists of three convolutional layers, several Residual Blocks^39^, two fractionally-strided convolutional layers with a stride of 1/2, and a final convolutional layer responsible for mapping features to RGB output. In our implementation, we perform our experiments with six and nine Residual Blocks (Table 2).

**Table 2:** Architecture and size of the activation maps of our implemented Generator for 6 Residual Blocks using a “channel_last” set-up.

Layer	Activation map size
Input	256 $\times$ 256 $\times$ 3
9 $\times$ 9 $\times$ 32 conv, stride 1	256 $\times$ 256 $\times$ 32
3 $\times$ 3 $\times$ 64 conv, stride 2	128 $\times$ 128 $\times$ 64
3 $\times$ 3 $\times$ 128 conv, stride 2	64 $\times$ 64 $\times$ 128
Residual block, 128 filters	64 $\times$ 64 $\times$ 128
Residual block, 128 filters	64 $\times$ 64 $\times$ 128
Residual block, 128 filters	64 $\times$ 64 $\times$ 128
Residual block, 128 filters	64 $\times$ 64 $\times$ 128
Residual block, 128 filters	64 $\times$ 64 $\times$ 128
Residual block, 128 filters	64 $\times$ 64 $\times$ 128
3 $\times$ 3 $\times$ 64 conv, stride 1/2	128 $\times$ 128 $\times$ 64
3 $\times$ 3 $\times$ 32 conv, stride 1/2	256 $\times$ 256 $\times$ 32
9 $\times$ 9 $\times$ 3 conv, stride 1	256 $\times$ 256 $\times$ 3

In contrast, for our discriminator networks, we employ 70 $\times$ 70 PatchGANs^28,40^. These PatchGANs are designed to classify whether 70 $\times$ 70 overlapping image patches are real or fake. This patch-level discriminator architecture boasts fewer parameters compared to a full-image discriminator and operates efficiently on images of varying sizes, leveraging a fully convolutional approach^28^.

#### ResNet type generators

The Generators *G* and *F* in our model are designed based upon the ResNet architecture. The ResNet architecture, similar to the U-Net architecture mentioned in the study by Zhu et al.^29^, incorporates skip-connections that enable the flow of information between non-consecutive layers.

It also features “Residual Blocks” between the Encoder and Decoder components. These Residual Blocks allow for the addition of input data to the block, facilitating the transfer of relevant information to the output of the layer. The addition operation is performed element-wise, and in cases where the dimensions of the input and output of the convolutional block do not match, techniques such as zero-filling or 1 $\times$ 1 convolutions can be employed to adjust the dimensions accordingly^39^.

Despite being introduced in 2015, the ResNet architecture remains widely used due to its effectiveness. The incorporation of Residual Blocks promotes gradient back-propagation during training, effectively preventing the issue of gradient vanishing as the network depth increases. Additionally, the computational cost of training a ResNet architecture with increasing layers is lower compared to the U-Net architecture. These advantages have led us to select the ResNet architecture for our work.

### PatchGAN type discriminators

The structure chosen for the Discriminators ($D_X$ and $D_Y$) is a PatchGAN. It is a CNN whose purpose is to perform a binary classification of an input image to decide whether it is real (1) or fictitious (0). However, unlike other CNNs, to make this decision, the PatchGAN architecture does not evaluate the entire input image, but segments it into N ${\times }$ N patches. During the training phase, the PatchGAN network evaluates the content of each of these patches to decide whether they are real or fictitious. Subsequently, the average of all the results is calculated to generate a final verdict on the complete image^41^.

The use of patch-based analysis in the discriminator has several advantages. First, it allows for more localized style evaluation, as different patches may exhibit variations in style within a single image. Second, it encourages the generator to produce visually convincing results at a fine-grained level, leading to sharper and more realistic outputs. Lastly, the patch-based approach reduces the computational complexity compared to evaluating the entire image, making it more efficient for training and inference^24^.

### Loss functions

In a Cycle GANs model the Generator Loss is calculated from three components: (i) *Adversary Loss* ($\alpha$); (ii) *Cycle Consistency Loss* ($\lambda$) and (iii) *Identity Loss* ($\delta$)^24^.

The first component, *Adversary Loss*, contains information to answer the following question: do the images created by each Generator manage to deceive the corresponding Discriminator? To calculate this, it is enough to use the *binary cross-entropy* function, since we must compare a tensor formed only by 1’s with the tensor of predictions made by the Discriminator.

The second component, *Cycle Consistency Loss*, evaluates the goodness of the Generators under the assumption of a complete cycle, i.e., if we apply the Generator *G* to an image of the source domain *X* (photograph) and afterwards we apply the Generator *F* to the generated fictitious image of the target domain *Y*, are we getting back to the original image? The smaller the difference between the source photograph and the reconstructed photograph, the more effective both Generators will be. The Mean Absolute Error (MAE) or L1 has been used to compare both images (source photograph and reconstructed photograph).

Finally, the third component, *Identity Loss*, tries to evaluate the goodness of the Generators in the following way: if the Generator *G* is fed with an image that already belongs to the target domain *Y*, will it keep it unchanged when it realises that it already belongs to the target domain? The smaller the difference between the input image and the output image, the more efficient the Generator will be. The L1 function has also been used to compare the two images.

A correct weighting of the three components mentioned above is essential for the generation of an efficient model. The calculation of the Discriminator Loss is simpler than the calculation of the Generator Loss. As in the *vanilla* GANs, we calculate the *Mean Squared Error* (MSE) of a real image and a fictitious image belonging to the same domain. The final loss of the Discriminators will be the mean of the previous ones.

### Top-k training of the proposed system

As discussed previously, the key to the study by Sinha *et al.* is to use during training only those images that have been able to “fool” the Discriminator most effectively. In this way, the Generator weights are updated using only the loss generated by those images^12^. The value proposition of the present work consists of applying this training scheme to a Cycle GANs system, consisting of two Generators and two Discriminators. To date, this approach has only been applied to linear GAN-type schemes, consisting of only one Generator and one Discriminator.

The update of the weights of the *G* Generator is done by taking into account the *k* images that it has created and that have been able to “confuse” the $D_Y$ Discriminator most effectively. The same procedure is applied for the training of the *F* Generator, but in this case taking into account the loss incurred by the $D_X$ Discriminator.

It should be mentioned that, during the first iterations of training, the predictions made by the Discriminators are random. This implies that their ability to decide whether the input image is a real image or not is quite limited. Therefore, it makes no sense to discard images from the *batch* during these first *epochs*. In the proposed system, training is initiated by using all the *batch* images to update the model weights. However, as both Generators and Discriminators learn, the proportion of *batch* images used is gradually reduced following an annealing schedule.

On the other hand, as stated by Sinha et al., it is not recommended to reduce the value of *k* to the point of selecting a single item from the *batch*, since this would dilute the effectiveness of using a sample of the training set^12^. We initially set *k* equal to the full batch size, at the beginning of training. As training progresses, we systematically decrease *k* by a constant factor, $\gamma$, with each epoch, until it reaches a minimum value of $k = \nu$, thus ensuring that *k* will never progress to the point of having only one element in the mini-batch. Equation (1) shows the calculation of *k* at each *epoch*:

1 $$\begin{aligned} k \leftarrow max(\gamma k, \nu ) \end{aligned}$$

*w*here $\gamma$ represents the decay factor and $\nu$ the minimum number of elements in the mini-batch.

Once the *k* value is calculated, it is used to choose the top-*k* images that are most effective in “deceiving” the Discriminators. As we explained previously, the component that captures the ability of the Generators to “deceive” the Discriminators is known as *Adversarial Loss*. In this way, once the Discriminators have issued their verdict on whether the input images are fictitious or not, we choose the images with a higher probability of being real (the closer the probability is to 1, the more effective the image has been in “fooling” the Discriminator). Then, we apply a mask to the calculation of the *Adversarial Loss* to take into account in the loss calculation only the referred *k* images.

## Results

### System evaluation

Once the Cycle GAN model described above was developed, it was trained following two approaches: (i) the first one, which we call basic or *vanilla*, is the one used by the scientific community to date and consists of updating the weights of the Generators taking into account all the samples in the batch and (ii) the second approach, based on the proposed top-*k* methodology, only takes into account for the update of the weights those *k* images that are able to maximise the “deception” of the respective Discriminators.

### Quantitative evaluation

In this section we show the quantitative and qualitative results achieved by both training approaches.

#### Generators and discriminators loss

Prior to training the Cycle GAN model using both methodologies, the PyTorch *Ray Tune* library was used to detect the optimal hyper-parameter combination in each case. “Optimal” is understood as that combination that minimises *G* and *F* Generator Loss and, at the same time, maximises the Loss of the Discriminators $D_X$ and $D_Y$. With respect to the *vanilla* training methodology, we defined a search space in *Ray Tune* for several hyper-parameters that affect model performance (see Tables 3 and 4).

Table 3 shows the optimal combinations returned by *Ray Tune* for the basic training methodology, during a training of 200 *epochs*. In particular, the last row shows the hyper-parameter combination that best satisfies this min-max game.

**Table 3:** Ray Tune results for *vanilla* training methodology.

Epochs	bs	#	$\alpha$	$\lambda$	$\delta$	*G*	*F*	$D_X$	$D_Y$
200	6	6	0.3	0.3	0.4	0.2009	0.1422	0.7113	0.6903
200	6	9	0.3	0.3	0.4	0.1551	0.1085	0.7848	0.8225
200	7	6	0.4	0.4	0.2	0.2116	0.1029	0.7546	0.7230
200	7	9	0.4	0.4	0.2	0.1269	0.1103	0.7848	0.7999

$\#$ stands for the number of Residual Blocks in the model, *bs* for the batch size and $\alpha$, $\lambda$ and $\delta$ for the weights of adversarial, cycle consistency and identity losses, respectively. Last four columns are for the losses of the generators *G* and *F* and the discriminators $D_X$ and $D_Y$.

Similarly, Table 4 shows the best combinations returned by *Ray Tune* for the top-*k* training methodology, with the last row being the optimal choice of hyper-parameters for a training of 200 *epochs*.

These results prove that the top-*k* methodology improves the behaviour of the loss functions offered by the *vanilla* training methodology, since we can observe how, after training both models for 200 *epochs*, the proposed method reaches lower loss levels for the Generators *G* and *F* while maintaining the loss levels of the Discriminators $D_X$ y $D_Y$ above those for the *vanilla* version.

**Table 4:** Ray Tune results for top-*k* training methodology.

Epochs	bs	#	$\alpha$	$\lambda$	$\delta$	$\gamma$	*G*	*F*	$D_X$	$D_Y$
200	7	6	0.3	0.3	0.4	0.99	0.2385	0.1995	0.7360	0.6714
						0.98	0.1983	0.1946	0.6306	0.6729
200	7	9	0.3	0.3	0.4	0.99	0.1578	0.1194	0.7818	0.7226
						0.98	0.1408	0.0890	0.7680	0.6612
200	7	6	0.4	0.4	0.2	0.99	0.0662	0.0619	0.7910	0.8038
						0.98	0.3250	0.1074	0.7788	0.7884
200	7	9	0.4	0.4	0.2	0.99	0.7238	0.0752	0.8941	1.1439
						0.98	0.1933	0.0664	0.8977	0.8398

$\#$ stands for the number of Residual Blocks in the model, *bs* for the batch size; $\alpha$, $\lambda$ and $\delta$ for the weights of adversarial, cycle consistency and identity losses, respectively; and $\gamma$ for the top-*k* decay. Last four columns are for the losses of the generators *G* and *F* and the discriminators $D_X$ and $D_Y$.

**Figure 6:**
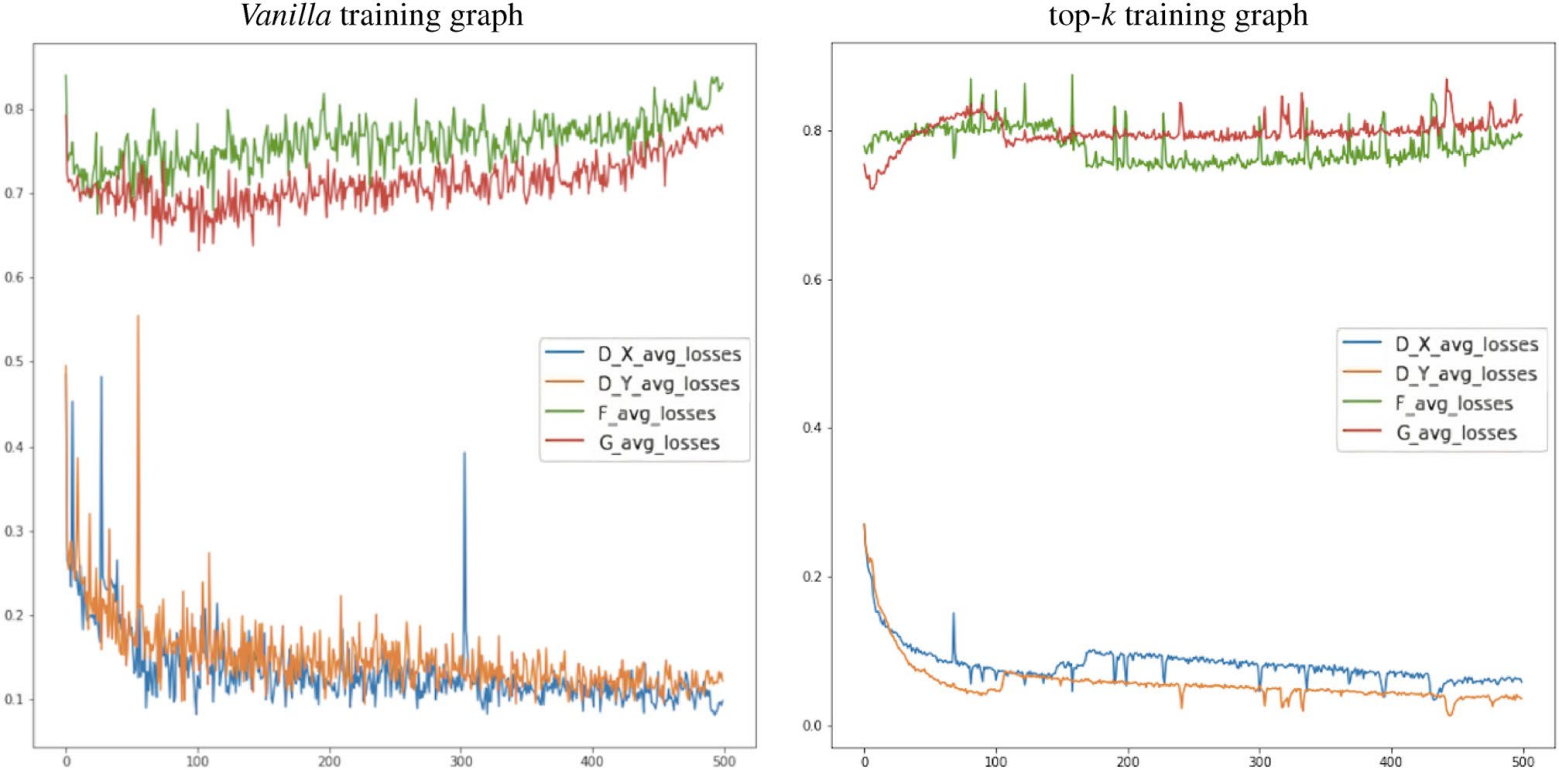
Loss curves for the *vanilla* and top-*k* trainings for 500 *epochs*.

To further validate these results, after identifying the optimal hyper-parameter combination, we trained both the *vanilla* and top-*k* methodologies for 500 *epochs*. Figure 6 shows the plots of the resulting loss curves obtained.

The training plots shown in Fig. 6 qualitatively support our previous findings. They prove that the top-*k* approach achieves lower levels of loss for Generator *G* (the one in charge of creating Monet frames) than in the basic training version. Regarding the Discriminator $D_Y$ (the one differentiating whether the generated image is really a work of Monet or not) both systems present similar levels. Furthermore, we can clearly observe that the top-*k* training approach achieves a more uniform and steady training, with its losses evolving smother than with the *vanilla* training approach. This leads to the conclusion that the choice of the top-*k* images in each iteration of the training facilitates and advances the convergence of training also in cyclic models. This statement matches the findings of Sinha et al. in their paper regarding training non-cyclic models.

#### Structural similarity measure index (SSMI)

The *Structural Similarity Measure Index* (SSMI) is a technique used to measure the similarity between two images. Specifically, the SSMI evaluates a test image *X* with respect to a reference image *Y* to quantify their visual similarity^42^. This metric was first introduced in the work of Zhou Wang et al. in early 2004 and was a turning point in the scientific community for the following reasons.

Most image comparison techniques used until 2004 were based on quantifying the difference between the absolute values of the individual pixels of the two images (using, for example, the mean square error). However, the SSMI metric proposes to assess the similarity of two images based on the following key characteristics: luminance, contrast and structure.

For a formal definition, we can define

$$\begin{aligned} l(x,y)=\frac{2\mu _x\mu _y+C_1}{\mu _x^2+\mu _y^2 + C_1} \end{aligned}$$

as the luminance similarity, where $\mu _x=\frac{1}{N}\sum ^N_{i=1}x_i$ and $C_1$ is a constant;

$$\begin{aligned} c(x,y)=\frac{2\sigma _x\sigma _y+C_2}{\sigma _x^2+\sigma _y^2 + C_2} \end{aligned}$$

as the constrast similarity, where $\sigma _x=(\frac{1}{N-1}\sum ^N_{i=1}(x_i - \mu _x)^2)^\frac{1}{2}$ and $C_2$ is a constant; and

$$\begin{aligned} s(x, y)=\frac{\sigma _{xy} + C_3}{\sigma _x\sigma _y + C_3} \end{aligned}$$

as the structural information, where $\sigma _x=\frac{1}{N-1}\sum ^N_{i=1}(x_i - \mu _x)(y_i - \mu _y)$ and $C_3$ is a constant. It is possible then to define the SSIM index as

$$\begin{aligned} SSIM(x,y)=l(x,y)\cdot c(x,y) \cdot s(x,y). \end{aligned}$$

We chose this metric since it attempts to mimic the human visual perception system for the first time. Our visual perceptual system is highly capable of identifying structural information in a scene and thus identifying differences between information extracted from a reference and a sample scene. Thus, a metric that replicates this behaviour performs best on tasks that involve differentiating between a sample and a reference image^43^.

In the present work, we employed the SSMI to compare the luminance and contrast between the input image (photograph) and the output image (Monet’s work). The aim was to compare whether the transformation implemented by the proposed system respects the structure and semantic content of the transformed photograph. The closer this index is to 1, the better the proposed system has succeeded in transforming the image while respecting the structure of the input image.

**Figure 7:**
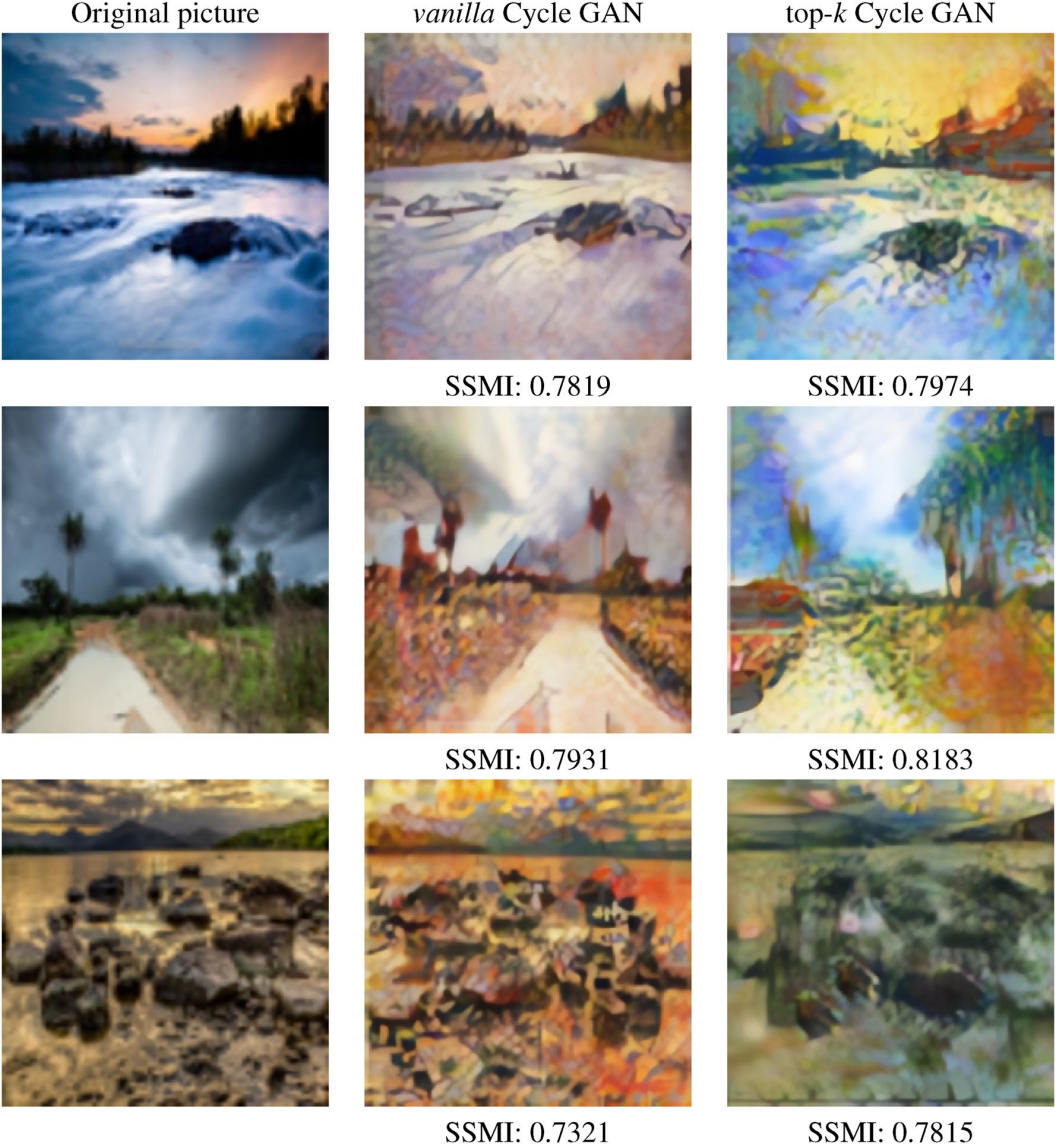
SSMI comparison between *vanilla* and top-*k* training.

As can be seen in Fig. 7, the SSMI obtained by comparing a photograph and a Monet artwork generated by the proposed system is higher than that calculated from the comparison between the photograph and the artwork generated with the *vanilla* Cycle GAN approach. This shows that, although both models manage to generate a Monet-style work quite satisfactorily, the proposed model respects the semantic and chromatic content of the input image to a greater extent.

### Qualitative evaluation

To further validate the results obtained we also carried out a visual assessment of the pictures generated with the proposed method. In this appraisal, images created using both the *vanilla* Cycle GAN and the proposed method were shown to people to be validated. This section analyses the questions asked, the basic characteristics of the groups surveyed and the main results obtained.

#### Visual assessment of the results obtained

As indicated in “System evaluation”, a hyper-parameter optimisation process was carried out for both methodologies: the Cycle GAN model in its *vanilla* training version and our proposed Cycle GAN top-*k* training method. As a result of that process, we chose the most efficient approach in quantitative terms.

**Figure 8:**
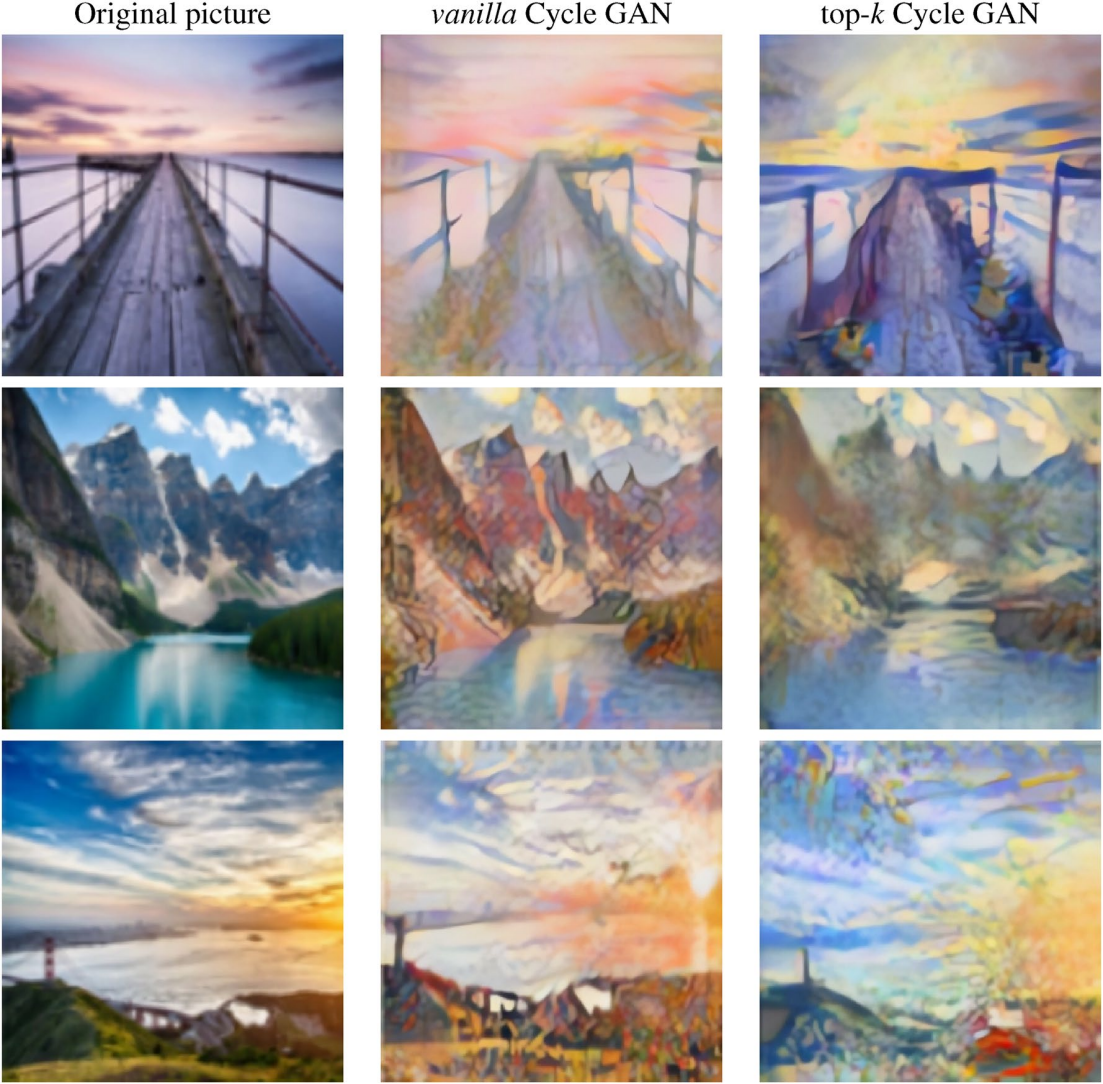
Sample of transformations. Picture to Monet.

**Figure 9:**
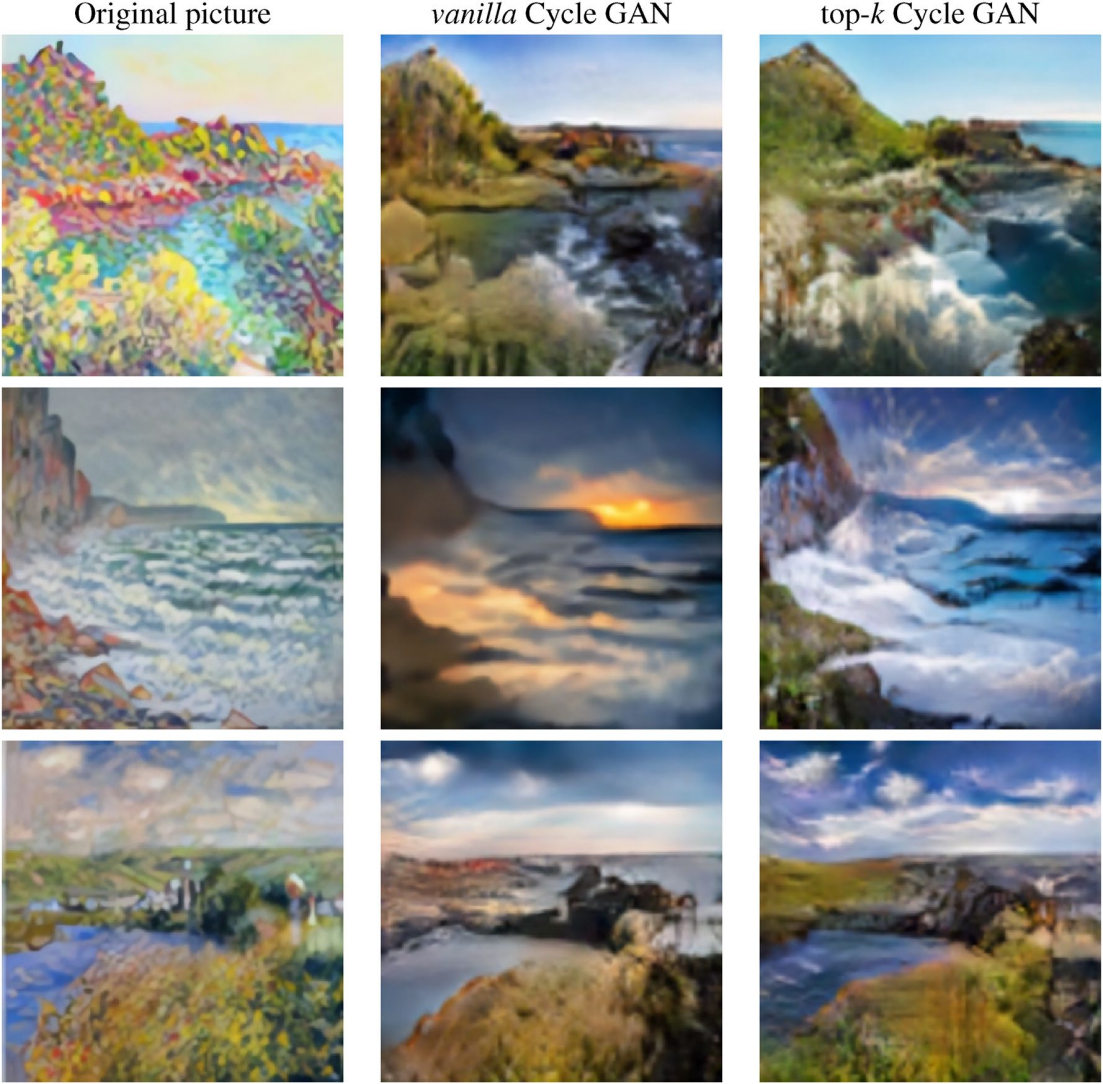
Sample of transformations. Monet to picture.

We then applied the final version of both models to a large sample of photographs with the aim of qualitatively evaluating the results obtained. Figure 8 shows several examples of the transformations achieved from picture to Monet by both methods. Similarly, Fig. 9 contains a sample of the transformations from Monet to picture.

As can be seen in the images above, both models recreate Monet’s artistic style quite well. To this end, they include strokes that imitate the painter’s brushstrokes and which, in turn, blur the edges of the objects to give the effect of the canvas. The tones of the photograph are also replaced by more vivid colours, frequently used in the artist’s works.

It should be noted, however, that the top-*k* approach is more successful in maintaining the chromatic and luminous structure of the photograph. Also, the images generated with the proposed system are less pixelated than those with *vanilla* system, which allows the human eye to be “fooled” more easily.

Based on the above qualitative observation, we can formulate the following hypotheses:

##### Hypothesis 1

The images generated, both with the *vanilla *methodology and with the proposed top-*k* system, are capable of being “mistaken” for real works by Monet.

##### Hypothesis 2

The proposed top-*k* system is a qualitative improvement over the basic training methodology of the Cycle GAN model.

#### Hypothesis-testing survey

In order to test the above-mentioned hypotheses, we carried out a survey by using the images generated with both models (*vanilla* and top-*k*). The survey, designed with Google Forms tool, consists of only five questions and was distributed between two clearly differentiated groups: the General Group and the Expert Group.

On the one hand, the General Group is composed by individuals with varied socio-demographic profiles. There is variability in the age of the respondents, in their level of education and place of residence. However, what characterises all respondents in this group is a lack of prior knowledge of the art sector. As the aim is to make the survey as widely available as possible, it has been shared through various social networks. Specifically, the link to the questionnaire was published on Whatsapp, Facebook and LinkedIn. In addition, we generated a QR code pointing to the survey to help the distribution of the survey. The questionnaire is available in Suppl Annex 1.

Following the distribution of the survey to the General Group through the indicated means of dissemination, a total of 100 responses were obtained. As an example of the results obtained, for question 2, respondents were asked to choose the image that, in their opinion, Monet could have painted, visualising a specific landscape. 61% of respondents chose “Option 2” (image generated by the proposed top-*k* system), as opposed to “Option 1” (image generated by the *vanilla* Cycle GAN system). This allows us to validate the veracity of Hypothesis 2, i.e. that the proposed system qualitatively improves the *vanilla* system.

On the other hand, the Group of Experts consisted of specialists in the visual arts. These were individuals with a degree in Art History and who form part of the staff of the *Museo del Prado* and *Museo Reina Sofía*, both located in Madrid, Spain. Specifically, the respondents belonged to the Visitor Services Department, Room Assistants and Curators. Since the profile defined in this case was clearly unique, the survey was distributed individually to each respondent by sending a unique link to the survey. In this case, 16 expert responses were received.

In this case, the responses obtained reveal that only 31% of respondents identified Monet’s original among several *fake* images generated by both the vanilla and top-*k* approach. This point reaffirms both Hypothesis 1 and Hypothesis 2, as the works generated by both systems are confused with the original works and, in turn, the quality of the images generated by the proposed system “mislead” the experts criteria.

## Discussion

In this work, we proposed a novel training methodology applied to Cycle GAN models based on updating the weights of the Generators taking into account only the the *k* images that were able to best fool their respective Discriminators. The results obtained with both the *vanilla* version and our proposed methodology were evaluated from a quantitative and qualitative perspective. The qualitative appraisal is particularly relevant given the artistic nature of the images produced.

As for the quantitative perspective, we showed that the use of the top-*k* methodology facilitates the convergence of the model and, in turn, improves the levels achieved by the loss functions of the Generators and Discriminators of the model.

Also in quantitative terms, the SSMI served to parameterise the similarity of the images generated, fulfilling the initial objective of generating images that maintain the semantic content, but imitating the artist’s style. In this sense, we found that the SSMI calculated from the images generated by the top-*k* system was closer to 1 than that calculated from the basic methodology. This shows that the proposed system maintains the semantic and chromatic content of the input image to a greater extent.

From a qualitative perspective and based on an initial visual assessment of the transformations achieved, two hypotheses were formulated: the first hypothesis stated that both the basic methodology and the proposed top-*k* methodology were capable of generating images that could be “mistaken” for real works of art. The second hypothesis stated that the proposed top-*k* system was a qualitative improvement over the basic training methodology of the Cycle GAN model. To test these hypotheses, we carried out a survey among two groups: the General Group and the Expert Group. The responses from the two samples confirmed both hypotheses, so we can conclude that there is evidence to affirm that the proposed methodology improves the basic training of the model in qualitative terms.

However, our work is based on two key assumptions. On the one hand, we assume that our expert group is representative, and their criteria are valid for generalizing their opinions. On the other hand, we specifically focus on a single painter’s style, and further experiments are required to test our method on different artistic styles.

Additionally, our work has some limitations. Firstly, the resolution of the images used is limited, which may result in some artifacts that are not easily noticeable to the human eye. Secondly, our second assumption regarding the focus on a single style also serves as a limitation, as we have not explored different pictorial styles, which remains an area for future research. Finally, it is important to note that the model employed in our study is intentionally a basic model, as our main objective was to demonstrate the improvement achieved through the implementation of the top-k training approach. Further investigations should explore more advanced and powerful methods in this domain.

As a final reflection, it should be noted that the ability of deep learning models to merge two distinct styles (photographer and painter) opens the door to new forms of artistic creation. However, it should be stressed that this artistic capacity of EAI does not detract from our creative capacity as human beings, nor does it seek to replace it. The reason is simple: the implemented AI systems can imitate pictorial tendencies from photographs, but both sources have to exist beforehand.

In this way, it is desirable that society interprets the artificial intelligence as a tool that seeks to complement the most valuable human capacity to generate new ideas or concepts: creativity.

### Supplementary Information

Supplementary Information.

## Data Availability

The datasets generated and/or analyzed during the current study are available in the monet2photo repository: https://www.tensorflow.org/datasets/catalog/cycle_gan#cycle_ganmonet2photo.

## References

[1] Santos I, Castro L, Rodriguez-Fernandez N, Torrente-Patino A, Carballal A (2021). Artificial neural networks and deep learning in the visual arts: A review. Neural Comput. Appl..

[2] Cohen H (1995). The further exploits of Aaron, painter. Stanford Hum. Rev..

[3] Arte e inteligencia artificial: Cuando los androides sueñan con crear $${\vert }$$ revista de verano $${\vert }$$ El País.

[4] Kalitina N (2019). Claude Monet.

[5] Callen A (2015). The Work of Art: Plein Air Painting and Artistic Identity in Nineteenth-Century France.

[6] Gupta, P. *Creating Art with Deep Learning*. https://medium.com/@prakhargupta_88888/arts-with-deep-learning-426b94b9f11e. Accessed July 2013.

[7] Tomei, M., Cornia, M., Baraldi, L. & Cucchiara, R. Art2real: Unfolding the reality of artworks via semantically-aware image-to-image translation. In *Proceedings of the IEEE/CVF Conference on Computer Vision and Pattern Recognition*. 5849–5859 (2019).

[8] Kotovenko, D., Sanakoyeu, A., Ma, P., Lang, S. & Ommer, B. A content transformation block for image style transfer. In *Proceedings of the IEEE/CVF Conference on Computer Vision and Pattern Recognition*. 10032–10041 (2019).

[9] Kotovenko, D., Wright, M., Heimbrecht, A. & Ommer, B. Rethinking style transfer: From pixels to parameterized brushstrokes. In *Proceedings of the IEEE/CVF Conference on Computer Vision and Pattern Recognition*. 12196–12205 (2021).

[10] Choi, J., Kim, S., Jeong, Y., Gwon, Y. & Yoon, S. Ilvr: Conditioning method for denoising diffusion probabilistic models. In *2021 IEEE, CVF International Conference on Computer Vision (ICCV)*. 14347–14356 (2021).

[11] Zhang, Y. *et al.* Inversion-based creativity transfer with diffusion models. *arXiv preprint* arXiv:2211.13203 (2022).

[12] Isinha S, Zhao Z, Alias Parth Goyal AG, Raffel CA, Odena A (2020). Top-k training of GANS: Improving GAN performance by throwing away bad samples. Adv. Neural Inf. Process. Syst..

[13] Gooch, B. & Gooch, A. *Non-Photorealistic Rendering* (CRC Press) (Google-Books-ID: AWG1DwAAQBAJ).

[14] Wu J (2014). Use of non-photorealistic rendering and photometric stereo in making bas-reliefs from photographs. Graph. Models.

[15] Gatys, L. A., Ecker, A. S. & Bethge, M. A neural algorithm of artistic style. *arXiv preprint* arXiv:1508.06576 (2015).

[16] Johnson, J., Alahi, A. & Fei-Fei, L. Perceptual losses for real-time style transfer and super-resolution. In *European Conference on Computer Vision*. 694–711 (Springer, 2016).

[17] Ulyanov, D., Lebedev, V., Vedaldi, A. & Lempitsky, V. S. Texture networks: Feed-forward synthesis of textures and stylized images. In *ICML*. Vol. 1(2). 4 (2016).

[18] Yin, R. Content aware neural style transfer. *arXiv preprint* arXiv:1601.04568 (2016).

[19] Liu, S. *et al.* Adaattn: Revisit attention mechanism in arbitrary neural style transfer. In *Proceedings of the IEEE/CVF International Conference on Computer Vision*. 6649–6658 (2021).

[20] An, J. *et al.* Artflow: Unbiased image style transfer via reversible neural flows. In *Proceedings of the IEEE/CVF Conference on Computer Vision and Pattern Recognition*. 862–871 (2021).

[21] Fu, T.-J., Wang, X. E. & Wang, W. Y. Language-driven artistic style transfer. In *European Conference on Computer Vision*. 717–734 (Springer, 2022).

[22] Yang, S., Hwang, H. & Ye, J. C. Zero-shot contrastive loss for text-guided diffusion image style transfer. *arXiv preprint* arXiv:2303.08622 (2023).

[23] Arora, S., Ge, R., Liang, Y., Ma, T. & Zhang, Y. Generalization and equilibrium in generative adversarial nets (GANS). In *International Conference on Machine Learning*. 224–232 (PMLR, 2017).

[24] Foster, D. *Generative Deep Learning: Teaching Machines to Paint, Write, Compose, and Play* (O’Reilly Media, Inc., 2019).

[25] Arjovsky, M., Chintala, S. & Bottou, L. Wasserstein GAN. *arXiv preprint* arXiv:1701.07875 (2017).

[26] Wei, X., Gong, B., Liu, Z., Lu, W. & Wang, L. Improving the improved training of Wasserstein GANS: A consistency term and its dual effect. *arXiv preprint* arXiv:1803.01541 (2018).

[27] Perarnau, G., Van De Weijer, J., Raducanu, B. & Álvarez, J. M. Invertible conditional GANS for image editing. *arXiv preprint* arXiv:1611.06355 (2016).

[28] Isola, P., Zhu, J.-Y., Zhou, T. & Efros, A. A. Image-to-image translation with conditional adversarial networks. In *Proceedings of the IEEE Conference on Computer Vision and Pattern Recognition*. 1125–1134 (2017).

[29] Zhu, J.-Y., Park, T., Isola, P. & Efros, A. A. Unpaired image-to-image translation using cycle-consistent adversarial networks. In *Proceedings of the IEEE International Conference on Computer Vision*. 2223–2232 (2017).

[30] Chen H (2021). Artistic style transfer with internal–external learning and contrastive learning. Adv. Neural Inf. Process. Syst..

[31] Zhang, Y. *et al.* Domain enhanced arbitrary image style transfer via contrastive learning. In *ACM SIGGRAPH 2022 Conference Proceedings*. 1–8 (2022).

[32] Batziou E, Ioannidis K, Patras I, Vrochidis S, Kompatsiaris I (2023). Artistic neural style transfer using Cyclegan and Fabemd by adaptive information selection. Pattern Recognit. Lett..

[33] Wu, X., Hu, Z., Sheng, L. & Xu, D. Styleformer: Real-time arbitrary style transfer via parametric style composition. In *Proceedings of the IEEE/CVF International Conference on Computer Vision*. 14618–14627 (2021).

[34] Deng, Y. *et al.* Stytr2: Image style transfer with transformers. In *Proceedings of the IEEE/CVF Conference on Computer Vision and Pattern Recognition*. 11326–11336 (2022).

[35] Kwon, G. & Ye, J. C. Diffusion-based image translation using disentangled style and content representation. *arXiv preprint* arXiv:2209.15264 (2022).

[36] Zhang, Y. *et al.* Inversion-based style transfer with diffusion models. In *Proceedings of the IEEE/CVF Conference on Computer Vision and Pattern Recognition*. 10146–10156 (2023).

[37] Maerten, A.-S. & Soydaner, D. From paintbrush to pixel: A review of deep neural networks in AI-generated art. *arXiv preprint* arXiv:2302.10913 (2023).

[38] Google. Cycle_gan/monet2photo. https://www.tensorflow.org/datasets/catalog/cycle_gan#cycle_ganmonet2photo. Accessed 13 July 2023.

[39] He, K., Zhang, X., Ren, S. & Sun, J. Deep residual learning for image recognition. In *2016 IEEE Conference on Computer Vision and Pattern Recognition (CVPR)*. 770–778. 10.1109/CVPR.2016.90. ISSN: 1063-6919 (2016).

[40] Li, C. & Wand, M. Precomputed real-time texture synthesis with Markovian generative adversarial networks. In *European Conference on Computer Vision*. 702–716 (Springer, 2016).

[41] Assens, M., Giro-i Nieto, X., McGuinness, K. & O’Connor, N. E. Pathgan: Visual scanpath prediction with generative adversarial networks. In *Proceedings of the European Conference on Computer Vision (ECCV) Workshops* (2018).

[42] Larkin, K. G. Structural similarity index simplified: Is there really a simpler concept at the heart of image quality measurement? *arXiv preprint* arXiv:1503.06680 (2015).

[43] Wang Z, Bovik AC, Sheikh HR, Simoncelli EP (2004). Image quality assessment: From error visibility to structural similarity. IEEE Trans. Image Process..

