# Supplementary material for: Improving the quality of image generation in art with top-k training and cyclic generative methods
Source: Sci Rep. 2023 Oct 18;13:17764. doi: 10.1038/s41598-023-44289-y (PMC10584976; doi:10.1038/s41598-023-44289-y)
Supplement: Supplementary file 1 — Supplementary Information. [file 41598_2023_44289_MOESM1_ESM.pdf]

Annex 1. Expert group survey

The following figures show the questionnaire employed to assess the proposed method by asking the expert group to answer it. It consists of 5 questions that are shown in Figures 1, 2, 3, 4 and 5.

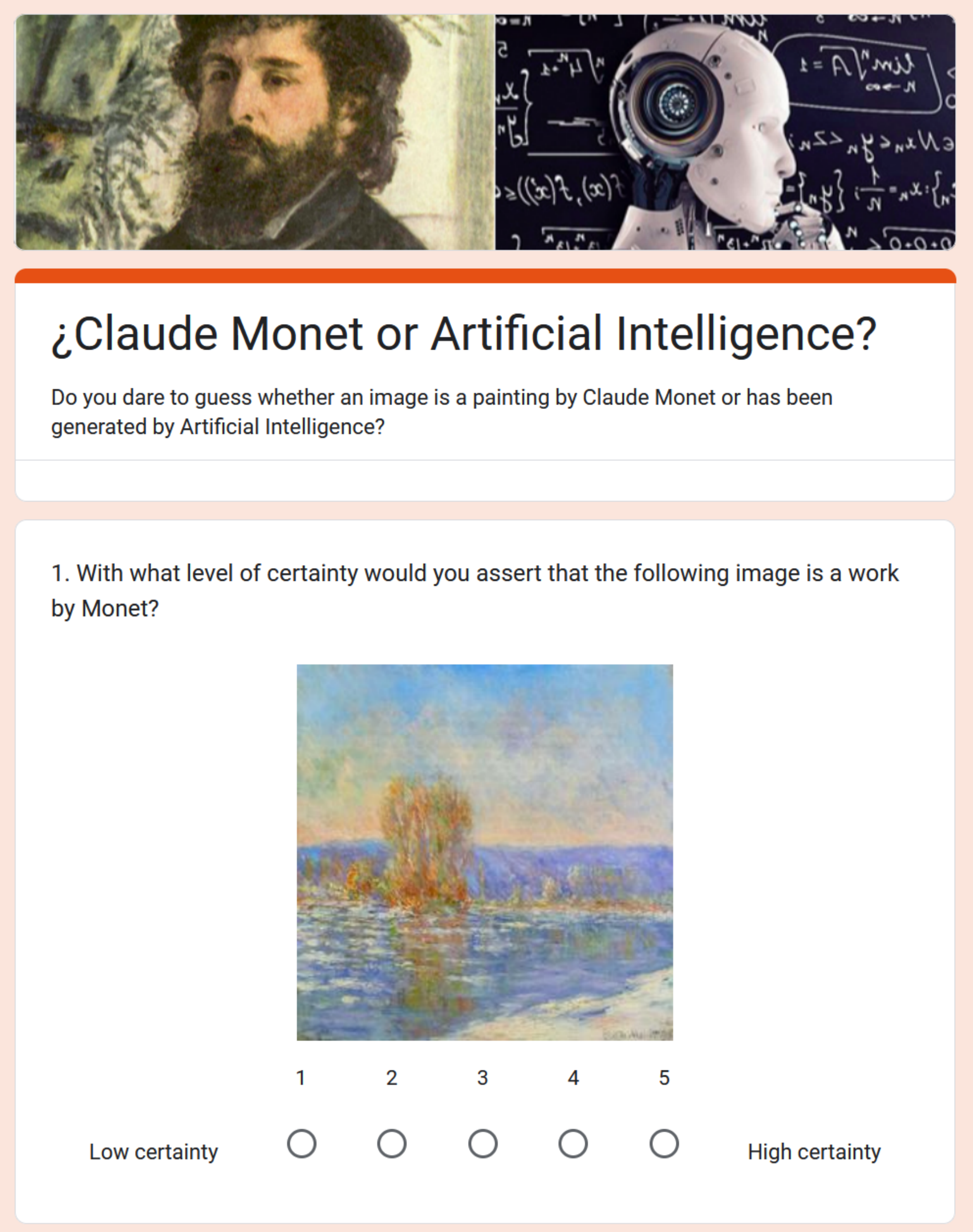

Figure 1. Experts group questionnaire. Question 1.

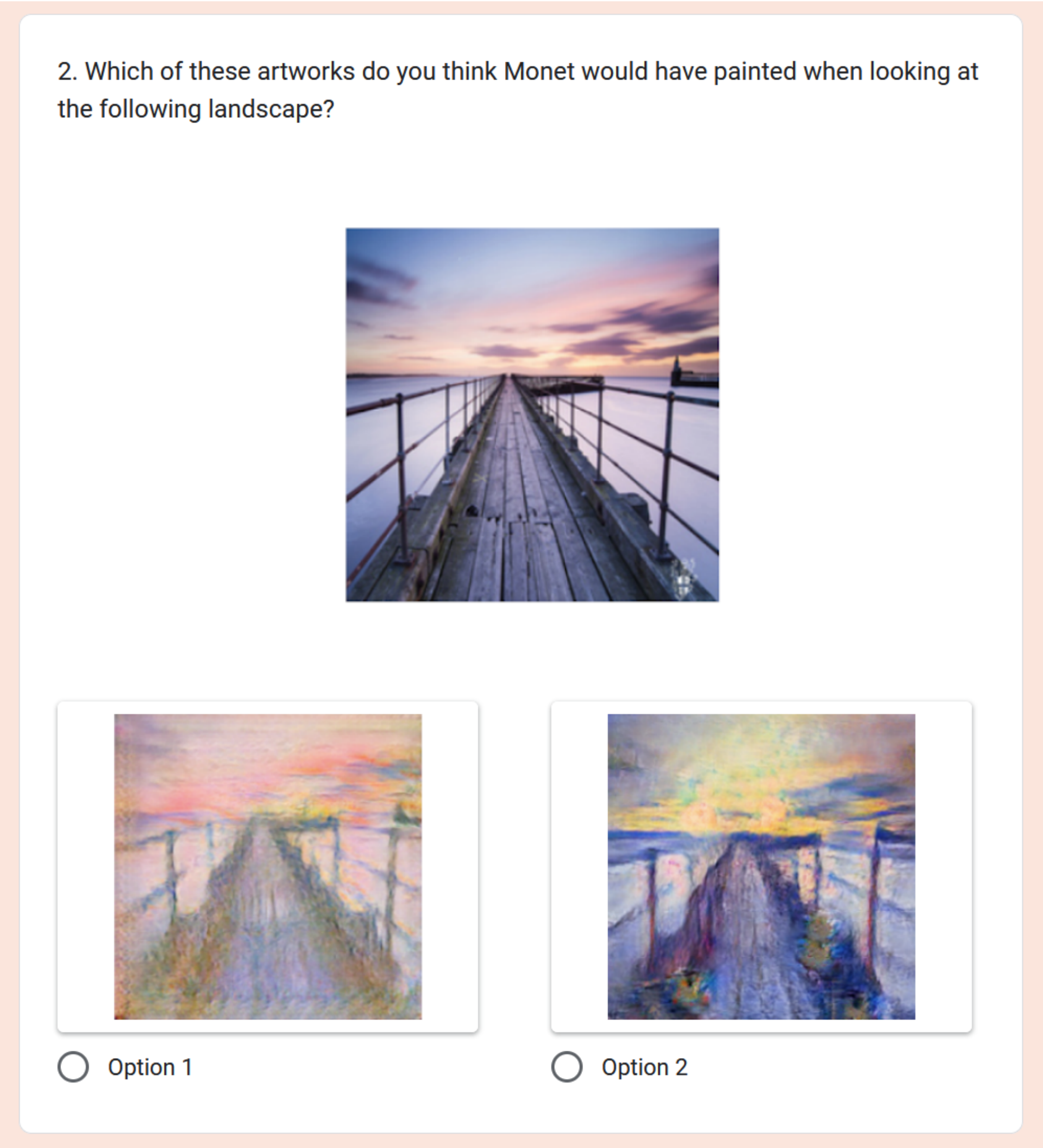

Figure 2. Experts group questionnaire. Question 2.

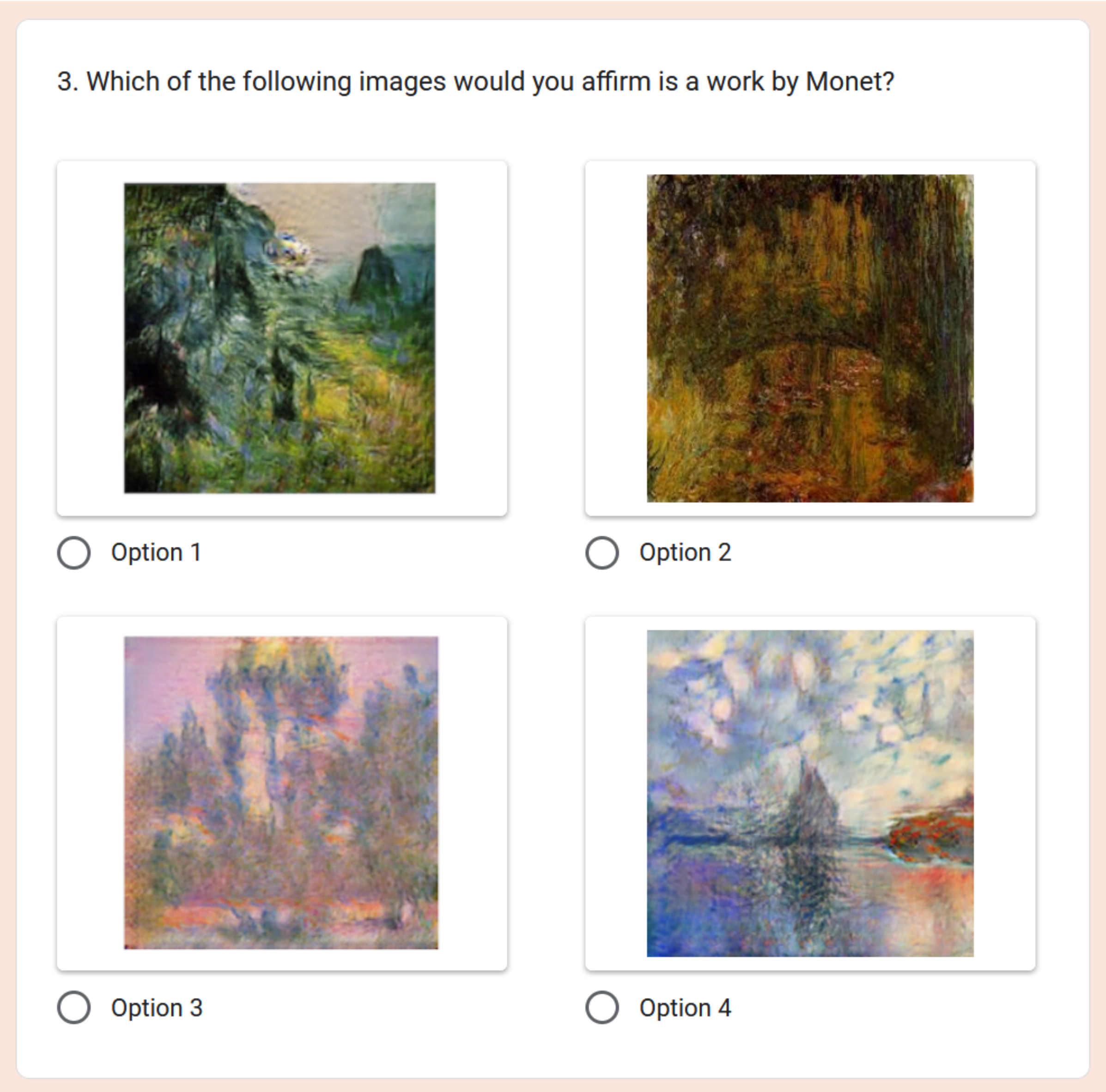

Figure 3. Experts group questionnaire. Question 3.

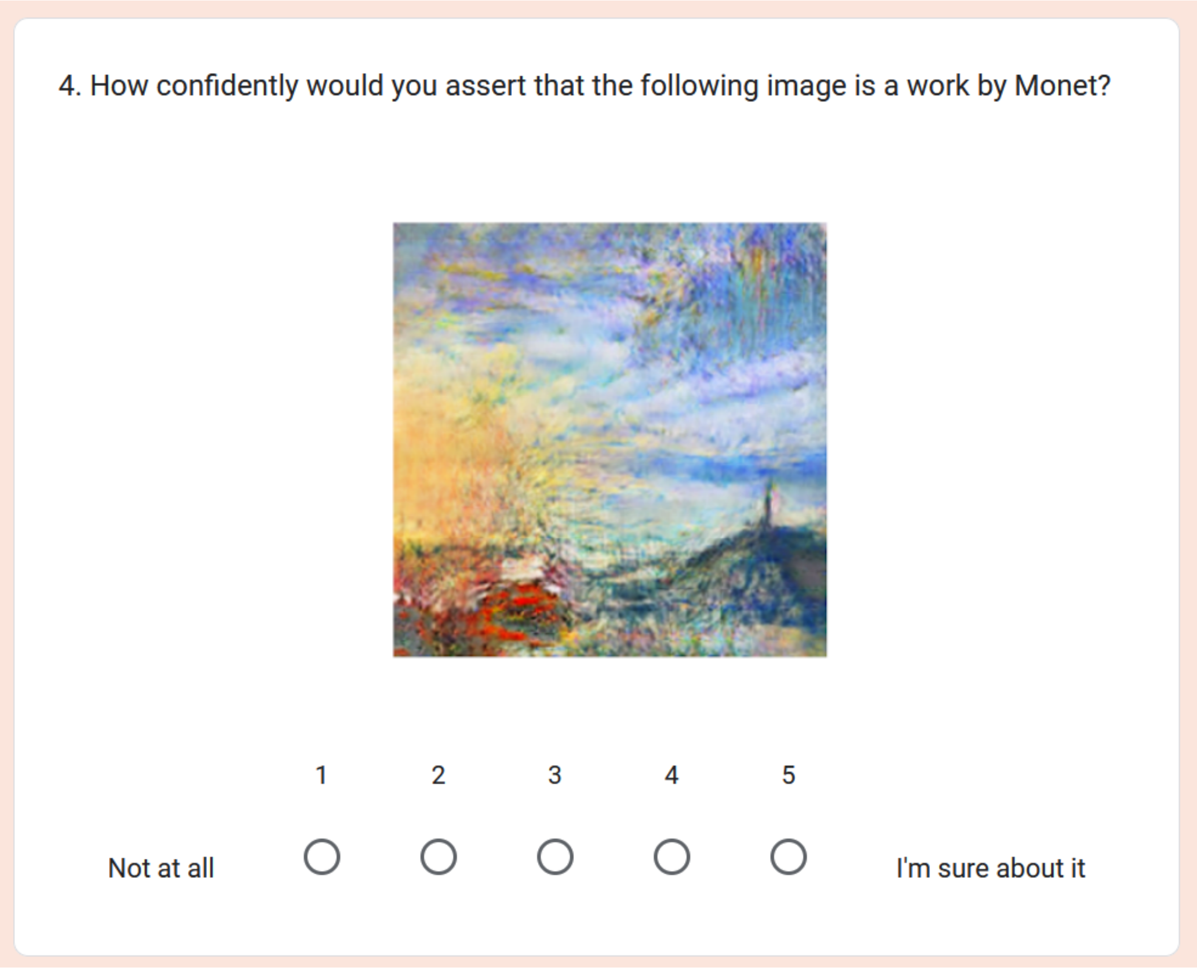

Figure 4. Experts group questionnaire. Question 4.

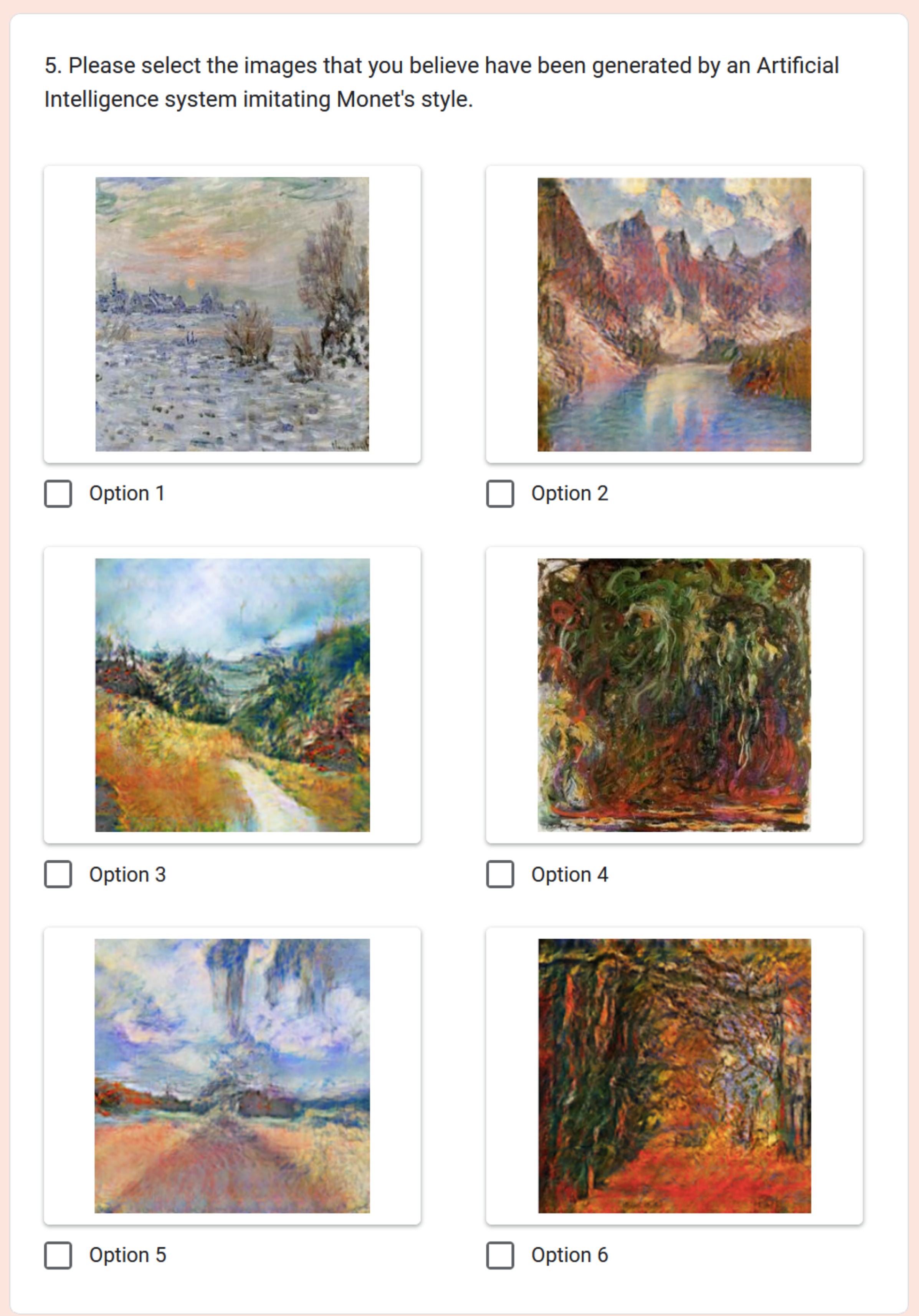

Figure 5. Experts group questionnaire. Question 5.
